# The first year of a new era

**DOI:** 10.7554/eLife.96413

**Published:** 2024-02-29

**Authors:** Timothy E Behrens, Yamini Dalal, Diane M Harper, Detlef Weigel

**Affiliations:** 1 eLife Cambridge United Kingdom

**Keywords:** scientific publishing, peer review, preprints, research assessment, research communication

## Abstract

What happened when eLife decided to eliminate accept/reject decisions after peer review?

One year ago, eLife made a radical change to the way that articles submitted to the journal were handled. Previously articles that had been selected for peer review were either accepted or rejected at the end of the review process. That all changed in January 2023: in future an article selected for peer review would be published on the eLife website as a Reviewed Preprint that included an eLife Assessment, Public Reviews and a response from the authors (if available). The eLife Assessment would be written by the editor and the reviewers, using a common vocabulary to summarise the significance of the findings and the strength of the evidence reported in the article. Moreover, we would continue to only review articles that were available as preprints. One of our aims was to give authors more control over the publication process (Eisen et al., 2022).

A lot has happened over the past year but, for us, the highlights have been that thousands of authors have put their trust in these new publishing ideas, and that our editors and reviewers have invested their time and energy to make the new system work. Together we have shown that a system can succeed in which scientific decisions are rich and nuanced; where a reviewer’s job is to comment on the science, not defend the journal’s name; and where authors can engage in discussions with reviewers without fear of having to start again. Convincing the wider research community – notably grant, hiring and tenure panels – of the many benefits of this approach is now a priority for eLife.

It is perhaps worth remembering that when we went public with our plans, there were predictions in every direction. Some thought that our new publishing model was too risky and that authors would not submit their work. Others were sure that we would be flooded with low-quality articles – or that the opposite would happen and that only those researchers who had the most confidence in their work would submit to us. There were also worries that editors and reviewers would not want to be involved in a system where there was no accept/reject decision and where authors were under no obligation to revise the article in response to comments from reviewers.

A year on, the reality is a lot more encouraging. We received more than 6200 submissions to the new model in its first year of operation, with last month (January 2024) being the best to date. About a third of these have been reviewed in depth, which is comparable with the fraction selected for review under our previous model, and we estimate (based on the ratings for significance and strength of evidence) that the quality of submissions has not changed significantly. More information is available in this Inside eLife post on the first year of the new approach.

The numbers are encouraging but they only tell part of the story. For the decade after it was launched in 2012, eLife used a consultative peer-review process to arrive at accept/reject outcomes. Although this process had advantages over traditional approaches in which editors alone made the final decision to accept or reject an article, we were painfully aware that it was far from perfect. We still made accept/reject decisions, knowing that these were subjective. We still asked for experiments in revision, knowing that many were a matter of taste. And we knew that rejected authors would encounter more sets of reviews at new journals, until the dice rolled in their favour.

For the last year, we have not had to make these decisions, and it has been a genuine pleasure. Consultations between editors and reviewers have focused on summarising the strengths and weaknesses of an article, knowing that their views are open for debate. The exchange between authors and reviewers has been freed from the sword of Damocles hanging over the authors’ heads. Moreover, it has been exciting to see that the overwhelming majority of authors have revised their articles in response to the reviewer comments, resulting in what we believe is better science all around.

Some of the eLife Assessments we have published have been remarkable, and we hope that authors will include them alongside publications on their CVs and in grant applications. Who, for example, would not want to draw attention to expert reviewers summarising their work like this: “This fundamental study advances substantially our understanding of sound encoding at synapses between single inner hair cells of the mouse cochlea and spiral ganglion neurons. Dual patch-clamp recordings – a technical tour-de force – and careful data analysis provide compelling evidence that the functional heterogeneity of these synapses contributes to the diversity of spontaneous and sound-evoked firing by the neurons. The work will be of broad interest to scientists in the field of auditory neuroscience” (Jaime Tobón and Moser, 2023).

Others have been remarkable for different reasons. The eLife Assessment and Public Reviews for three articles about the hominin species *Homo naledi* have been widely read and were quoted in the subsequent press coverage (see, for example, Callaway, 2023). Such examples demonstrate the importance of nuanced public assessments of science in the digital era, especially for topics of broad interest.

The last 12 months have been a learning experience for us, and we will continue to adapt in the light of what we learn. For example, while we currently review only about one third of submissions, we are keen to see the eLife approach extended to articles that often do not clear the editorial hurdle at broad-interest journals, such as important negative results, or seemingly incomplete “stories” that nevertheless contain important findings. We very much welcome ideas on how to make progress towards this long-term aim. We are also keen to encourage other publishers to adopt at least some of aspects of the new eLife approach and, towards this end, the software infrastructure we are building to support the review and publication of preprints will be made freely available.

This past year has been an adventure on many fronts. The original driving force behind our new approach, Mike Eisen, was replaced as Editor-in-Chief last October. Going into the second year of this new era, we remain committed to our vision of reforming scientific publishing and reaffirming eLife’s ambition to be a force for positive change in science.
